# Mechanism Exploration of Amyloid-β-42 Disaggregation by Single-Chain Variable Fragments of Alzheimer’s Disease Therapeutic Antibodies

**DOI:** 10.3390/ijms24098371

**Published:** 2023-05-06

**Authors:** Xing Fan, Lipeng Xu, Jianhao Zhang, Yidan Wang, Zirui Wu, Wenjing Sun, Xin Yao, Xu Wang, Shanshan Guan, Yaming Shan

**Affiliations:** 1 National Engineering Laboratory for AIDS Vaccine, School of Life Sciences, Jilin University, Changchun 130012, China; fanxing9919@mails.jlu.edu.cn (X.F.); xulp22@mails.jlu.edu.cn (L.X.); zhangjh1319@mails.jlu.edu.cn (J.Z.); wuzr2021@jlu.edu.cn (Z.W.); sunwj1319@mails.jlu.edu.cn (W.S.); yaoxin21@mails.jlu.edu.cn (X.Y.); wangx22@mails.jlu.edu.cn (X.W.); 2 College of Biology and Food Engineering, Jilin Engineering Normal University, Changchun 130052, China; 3 Key Laboratory for Molecular Enzymology and Engineering, The Ministry of Education, School of Life Sciences, Jilin University, Changchun 130012, China

**Keywords:** Alzheimer’s disease, amyloid-β-42 protein, single-chain variable fragment, monoclonal antibody, molecular dynamics simulation

## Abstract

Alzheimer’s disease (AD) is a specific neurodegenerative disease. This study adopts single-chain variable fragments (scFvs) as a potential immunotherapeutic precursor for AD. According to the remarkable effects of monoclonal antibodies, such as the depolymerization or promotion of Aβ42 efflux by Crenezumab, Solanezumab, and 12B4, it is attractive to prepare corresponding scFvs targeting amyloid-β-42 protein (Aβ42) and investigate their biological activities. Crenezumab-like scFv (scFv-C), Solanezumab-like scFv (scFv-S), and 12B4-like scFv (scFv-12B4) were designed and constructed. The thermal stabilities and binding ability to Aβ42 of scFv-C, scFv-S, and scFv-12B4 were evaluated using unfolding profile and enzyme-linked immunosorbent assay. As the results indicated that scFv-C could recognize Aβ42 monomer/oligomer and promote the disaggregation of Aβ42 fiber as determined by the Thioflavin-T assay, the potential mechanism of its interaction with Aβ42 was investigated using molecular dynamics analysis. Interactions involving hydrogen bonds and salt bonds were predicted between scFv-C and Aβ42 pentamer, suggesting the possibility of inhibiting further aggregation of Aβ42. The successfully prepared scFvs, especially scFv-C, with favorable biological activity targeting Aβ42, might be developed for a potentially efficacious clinical application for AD.

## 1. Introduction

Alzheimer’s disease (AD) is a progressive neurodegenerative disease that impairs memory and cognitive judgment [1,2]. In 2019, the number of people living with AD worldwide was estimated at 50 million, and it was listed as the seventh-leading cause of death in 2021 [3]. In such a problematic situation, developing effective prevention methods, sensitive diagnostic methods, and effective treatment programs is urgent. Studies have shown three major pathological features in the brains of patients with AD: plaques formed by the accumulation of amyloid-β-42 protein (Aβ42), tangles formed by aggregation of highly phosphorylated microtubule-binding proteins, and neuron loss [4]. The “Aβ cascade hypothesis” has received the most acceptance, suggesting that soluble Aβ42 oligomer (AβO) is the main neurotoxin responsible for synaptic damage and neuronal death [5,6,7].

Passive immunotherapy targeting Aβ42 has emerged as one of the promising treatment strategies [8]. A few monoclonal antibodies (mAb) against Aβ42 have entered clinical trials or been approved for marketing [9]. Two phase III clinical trials of Bapineuzumab (NCT00574132 and NCT00575055), developed by Janssen and Pfizer, demonstrated that impaired cerebrovascular integrity occurs during anti-amyloid immunotherapy when amyloid is removed from the vessel wall [10]. Crenezumab, developed by Genentech, has an affinity for AβOs at least ten times that of Aβ42 monomers (0.4 to 0.6 vs. 3.0 to 5.0 nM), could block aggregation of monomers in vitro, and could induce depolymerization of existing aggregates [11]. Solanezumab, a humanized murine antibody, was tested in clinical phase I, II, and III studies. The results indicated that Solanezumab might have efficacy in AD through a significant effect on or promotion of Aβ42 efflux from the central nervous system to the peripheral circulation [12,13]. That analysis saw a trend to improve cognition with Solanezumab in people with mild AD but missed statistical significance [13]. MAb 12B4 targets the residues 3 to 7 of Aβ42, and it recognizes soluble monomers, oligomers, or insoluble aggregates of Aβ species [14].

A single-chain variable fragment (scFv) is a genetically engineered antibody that contains only the variable regions of the heavy and light chains of an antibody and is linked by flexible hinges [15]. Although anti-amyloid mAb that substantially removes Aβ42 plaques is associated with an adverse event known as amyloid-related imaging abnormalities (ARIA) with edema, microhemorrhage, or superficial siderosis [16], currently, radiologists can optimally impact the management of patients receiving targeted AD therapies [17]. Furthermore, compared with mAb, scFv has advantages, such as the low molecular weights of scFvs enhance the capacity to pass through the blood–brain barrier. ScFvs are safer than mAbs because scFvs do not contain a crystallizable fraction, which might activate microglia and trigger the complement system. Moreover, scFvs can be widely expressed in the prokaryotic expression system and are readily available [18,19].

In our previous studies, both a 12B4-like scFv (scFv-12B4) targeting Aβ42 and a thermophilic acylpeptide hydrolase were co-conjugated to gold nanorods. The results showed that the gold nanorod complex could reduce Aβ42-induced cytotoxicity both in vitro and in vivo [20].

Therefore, scFvs were designed as novel potential therapeutic precursors for AD in this study. Crenezumab-like scFv (scFv-C), Solanezumab-like scFv (scFv-S), and 12B4-like scFv (scFv-12B4) were designed based on the antigen-recognition sequences of corresponding monoclonal antibodies, and their biological activities were characterized. To explore the binding mechanism between scFv-C and Aβ42, molecular dynamics simulation was applied. Molecular dynamics simulation is a computer simulation method to study the physical movement of macromolecules such as proteins [21,22]. Reasonable initial models were constructed to explore the structure and energy changes of scFvs in exerting their functions by simulating the dynamic process [23,24]. This study might provide a potential therapeutic precursor for AD.

## 2. Results

### 2.1. Construction and Identification of pscFvs

**Figure 1:**
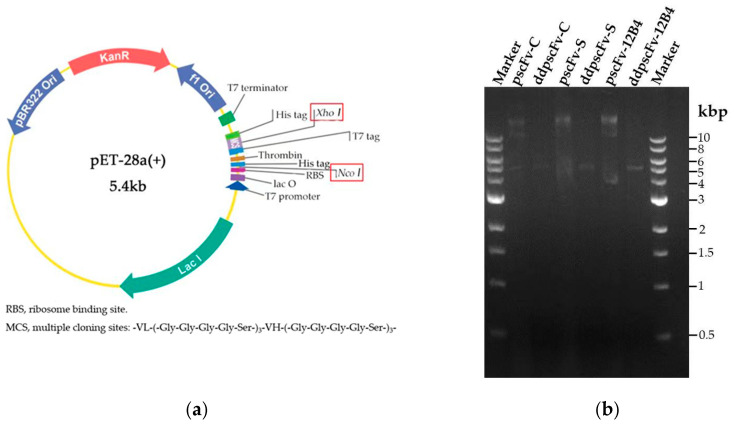
The construction and characterization of pscFvs: (**a**) the plasmid profile of pscFvs, and (**b**) double digestion of pscFvs.

The plasmids (Figure 1a) could be double digested by Nco I and Xho I, and the target band (~5 kb) could be observed after nucleic acid electrophoresis (Figure 1b). The sequencing results were also consistent with expectations, confirming the successful construction of the recombinant pscFvs.

### 2.2. Expression and Purification of scFvs

**Figure 2:**
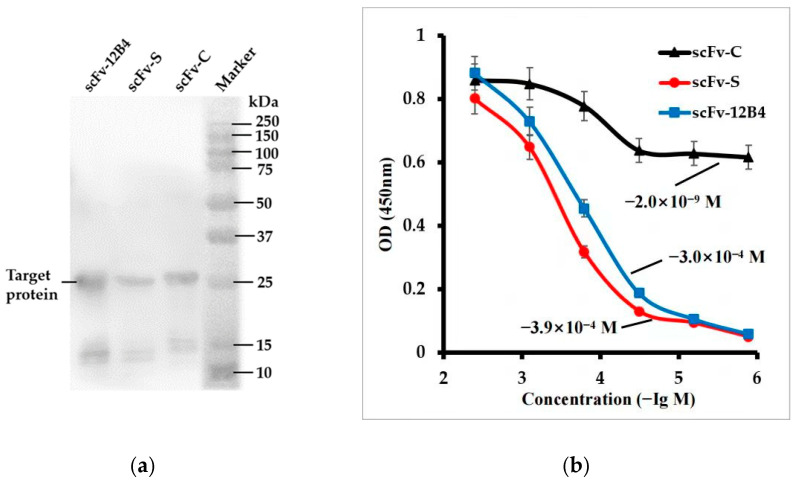
Characterization of scFvs activity: (**a**) Western blotting of scFvs, and (**b**) the binding activities of scFvs to Aβ42.

PscFvs were transformed into BL21(DE3) competent cells, and the ideal IPTG concentration for inducing protein expression is 1 mM at 37 °C for 4 h. The Western blot analysis confirmed the successful expression of scFvs in BL21(DE3) competent cells (Figure 2a).

### 2.3. scFv-C Could Give a Strong Binding Ability with Aβ42

The binding activities of purified scFv-C, scFv-S, or scFv-12B4 against Aβ42 were determined using ELISA (Figure 2b). The dissociation constant (Kd) corresponds to the scFv concentration at which half of the Aβ42 are ocupied at equilibrium [19]. The Kd value of scFv-C was ~2.0 × 10^−9^ M for Aβ42. The Kd value of scFv-S was ~3.9 × 10^−4^ M, and that of scFv-12B4 was ~3.0 × 10^−4^ M. The results indicate that scFv-C has a better binding ability than that of scFv-12B4 or scFv-S.

### 2.4. scFv Conformation Has Good Thermal Stability

**Figure 3:**
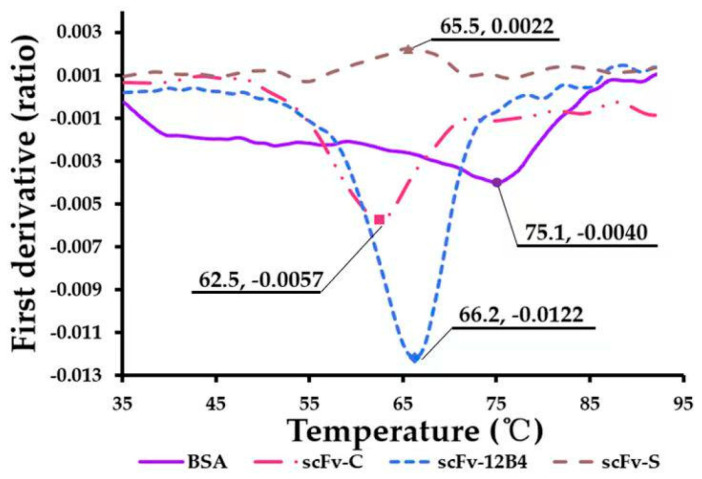
The thermal stability of scFvs.

The temperature tolerance of scFvs might allow them to stably recognize and clear Aβ42. The thermal stabilities of scFvs were evaluated using Nano Temper Tycho under an unfolding condition during the heating process from 35 °C to 95 °C (Figure 3). The results show that the conformations of scFvs could give specific thermal stability. The ratio of 350 nm/330 nm is a measure of the spectral shift of the fluorescence emission profile of tryptophan (Trp) residues, which is used to detect Trp residues exposed on the surface during the unfolding process. Therefore, before the deformation temperature, the conformation of scFvs remained relatively stable and could maintain a complete conformation binding with Aβ42.

### 2.5. scFvs Can Promote Depolymerization of Aggregated Aβ42

**Figure 4:**
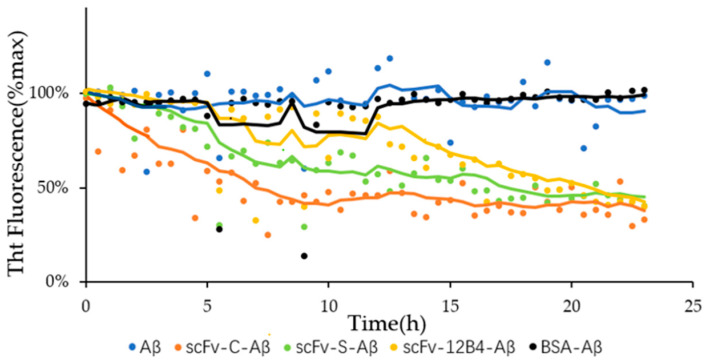
Depolymerization of aggregated Aβ42 by scFvs. The average of the points were plotted in corresponding color.

ScFvs can depolymerize aggregated Aβ42. After incubation for 23 h, the percentage of fluorescence intensity of the mixture treated with scFvs decreased. The results indicated that the amount of aggregated Aβ42 treated with scFv-C, scFv-S, and scFv-S dropped to 39%, 45%, and 42%, respectively (Figure 4), while the fluorescence intensity of aggregated Aβ42 dropped to 91%. The above results suggested that scFvs could depolymerize aggregated Aβ42.

### 2.6. Potential Interaction between scFv-C and Aβ_5_

The results indicated that scFv-C could give a better binding ability to Aβ42 than scFv-12B4 and scFv-S did; therefore, the potential binding modes between scFv-C and Aβ_5_, the Aβ42 pentamer, were further analyzed via MD. The results showed that the interactions between scFv-C and Aβ_5_ were possible through hydrogen bonds and salt bonds. The probabilities of hydrogen bonds of the residues located at the binding interface are shown in Table 1. The possibilities of most hydrogen bonds are between 9% and 20%, where the most considerable probability is the hydrogen bond between ASN178^scFv-C^ and GLN15^Aβ5-mono5^, at 22.77%.

**Figure 5:**
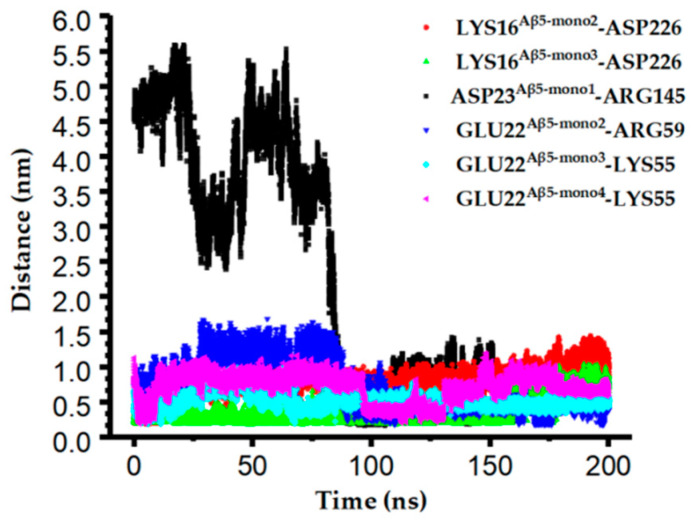
The distances of vital charged residues at the binding interface in the scFv-C-Aβ_5_ complex.

The monitoring of the distances of charged residues at the binding interface revealed that the shortest distances between the residues (Lys55^scFv-C^, Arg59^scFv-C^, Arg145^scFv-C^, and Asp226^scFv-C^) on scFv-C and the residues (Asp23^Aβ5-mono1^, Lys16^Aβ5-mono2^, Lys16^Aβ5-mono3^, Glu22^Aβ5-mono2^, Glu22^Aβ5-mono3^, and Glu22^Aβ5-mono4^) on Aβ_5_ can be as short as 0.2 nm, as shown in Figure 5 and Figure S2, which may favor salt bond formation.

**Figure 6:**
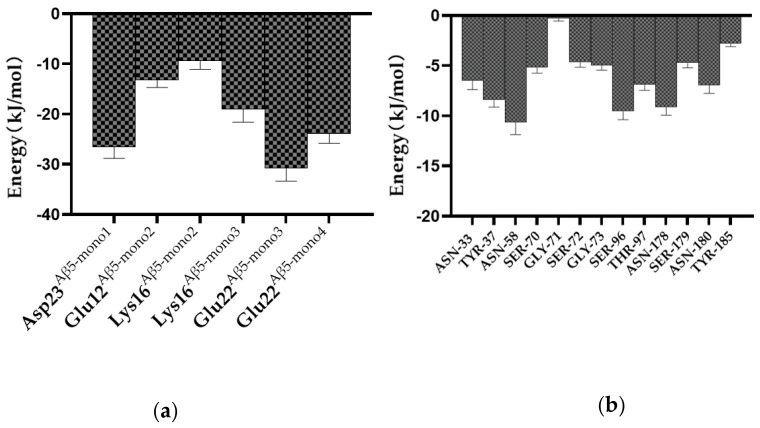
The binding energy of vital residues in scFv-C-Aβ_5_ complex: (**a**) energies of the residues from Aβ_5_ involved in salt-bridge formation, and (**b**) energies of the residues from scFv-C involved in H-bond formation. The error bars represent standard errors.

The binding potential of vital residues was monitored via MM/PBSA. Binding free energy represents the binding potential of residues, and the smaller the value, the stronger the binding potential. In Figure 6a, the binding free energy results indicate that six of the residues of Aβ_5_—Asp23^Aβ5-mono1^, Glu22^Aβ5-mono2^, Lys16^Aβ5-mono2^, Lys16^Aβ5-mono3^, Glu22^Aβ5-mono3^, and Glu22^Aβ5-mono4^—contribute more to the scFv-C and Aβ_5_ complex formation than other residues. The significant energetic contributions of the charged residues suggest that salt bonds might play dominant roles. The energy contributions of the residues of scFv-C involved in hydrogen bond formation with Aβ_5_ are shown in Figure 6b. Although the probabilities of hydrogen bonds are not high throughout the simulation, as seen in Table 1, the binding energies of the residues involved in hydrogen bonds still present favorable contributions to scFv-C and Aβ_5_ complex formation.

### 2.7. Simulated Structure Analysis of scFv-C-Aβ_5_ Complex and Aβ_5_ System

**Figure 7:**
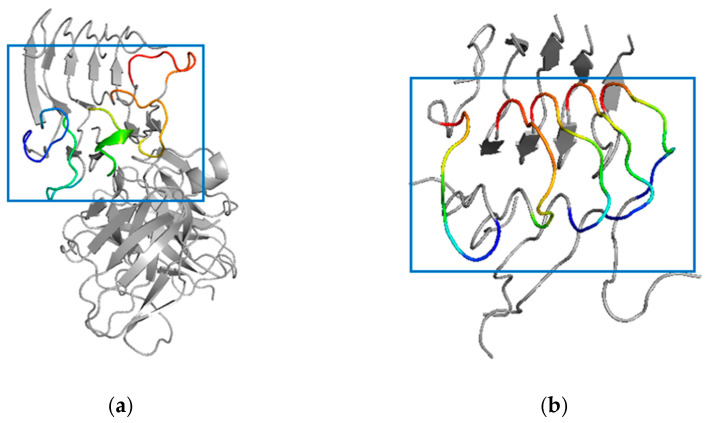
Simulated structure of the scFv-C-Aβ_5_ complex system: (**a**) structure of scFv-C-Aβ_5_ complex, and (**b**) binding surface of Aβ_5_.

In this part, the scFv-C-Aβ_5_ complex system and the Aβ_5_ system were compared after 200 ns simulation. The results show that after 200 ns simulation, the local disturbance of Aβ_5_ bound to scFv-C is apparent in the scFv-C-Aβ_5_ complex system. Notably, the residues Ala21 to Gly29 on each monomer of Aβ_5_, indicated by the blue box in Figure 7a, are disturbed. To determine the degree of structural deformation, the root-mean-square deviations (RMSD) of the simulated systems were calculated. RMSD could be employed to evaluate structural perturbation, and the higher the value, the stronger the perturbation.

**Figure 8:**
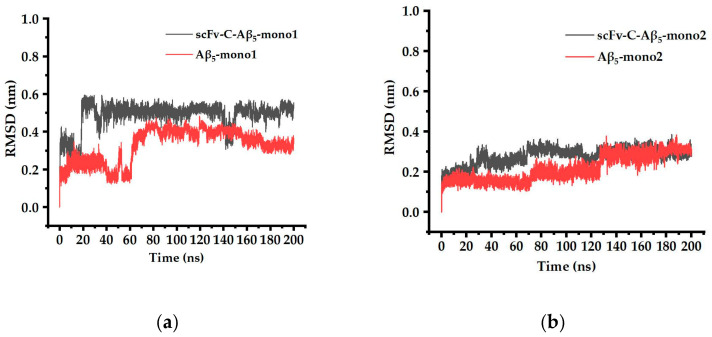

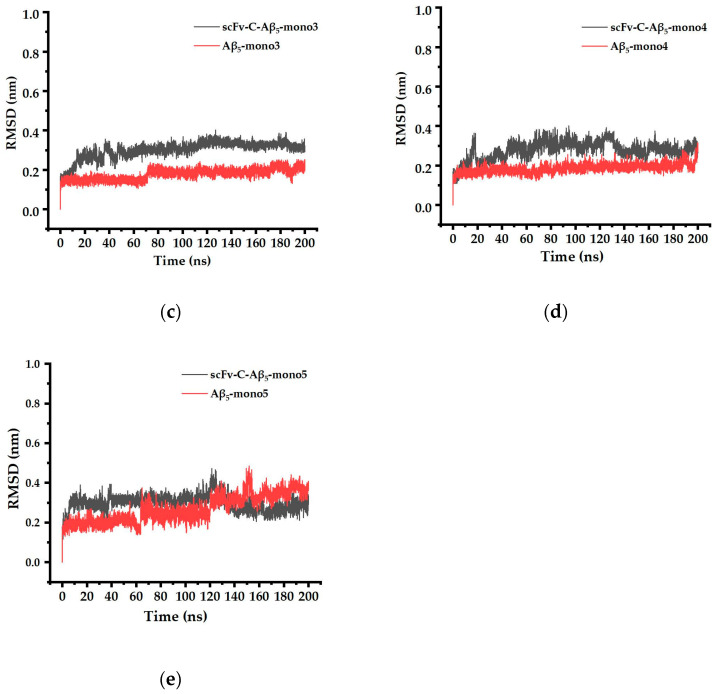
The RMSD of the Ala21 to Gly29 region of five Aβ monomers of bound and unbound scFv-C: (**a**) the RMSD data of scFv-C-Aβ_5_-mono1 and Aβ_5_-mono1; (**b**) the RMSD data of scFv-C-Aβ_5_-mono2 and Aβ_5_-mono2; (**c**) the RMSD data of scFv-C-Aβ_5_-mono3 and Aβ_5_-mono3; (**d**) the RMSD data of scFv-C-Aβ_5_-mono4 and Aβ_5_-mono4; and (**e**) the RMSD data of scFv-C-Aβ_5_-mono5 and Aβ_5_-mono5.

The RMSD of the Ala21 to Gly29 region of five Aβ monomers, bound and unbound to scFv-C, were obtained and compared. The results showed that the RMSD value of the Ala21 to Gly29 region of mono1, mono3, and mono4 of Aβ_5_ was significantly increased by antibody binding, indicating that scFv-C may have the potential to disrupt the structure of Aβ_5_ (Figure 8).

**Figure 9:**
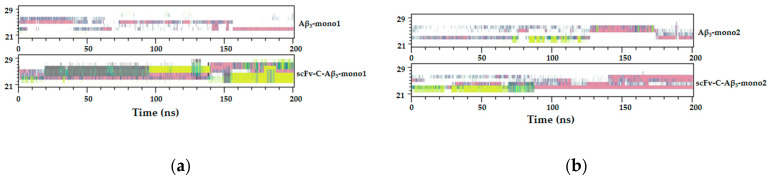

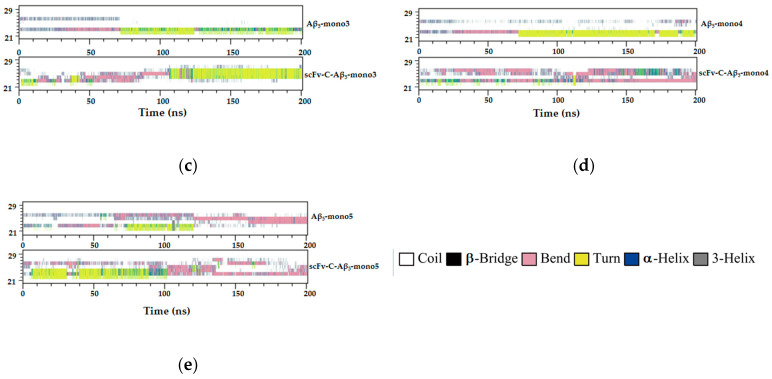
Structural transformation of the Ala21-Gly29 region of Aβ_5_ in bound and unbound scFv-C systems: (**a**) structural transformation of scFv-C-Aβ_5_-mono1 and Aβ_5_-mono1; (**b**) structural transformation of scFv-C-Aβ_5_-mono2 and Aβ_5_-mono2; (**c**) structural transformation of scFv-C-Aβ_5_-mono3 and Aβ_5_-mono3; (**d**) structural transformation of scFv-C-Aβ_5_-mono4 and Aβ_5_-mono4; and (**e**) structural transformation of scFv-C-Aβ_5_-mono5 and Aβ_5_-mono5.

The secondary structure of the Ala21 to Gly29 region was further monitored. The structural changes of the Ala21 to Gly29 region of Aβ_5_ in the scFv-C-Aβ5 complex system and individual Aβ5 system were analyzed (Figure 9). After comparing mono1 to mono5 between the bound and unbound systems, the conclusion is that the binding of scFv-C tends to deaggregate Aβ42 fiber.

## 3. Discussion

AD is a common neurodegenerative disease, with a rate of up to 50% to 70% of all dementia cases [25]. ScFv, in particular, attaches easily to AβOs and fibrils. The passive immune response of scFv with a low molecular weight, is advantageous for crossing the blood–brain barrier [18,19]. Previous studies have indicated that Aβ42 monomers may be of physiological significance to nerve cells in healthy individuals [19]. An antibody that exclusively recognizes oligomers and fibrils, rather than monomers, is an ideal agent for AD treatment [20].

Three mAbs, Crenezumab, Solanezumab, and 12B4, were chosen in this study. 12B4 displayed the most substantial binding ability to polypeptides and the weakest binding ability to AβOs. The reason might be that 12B4 could recognize residues 3 to 7 of Aβ42. After its aggregation to form oligomers or fibers, the N-terminal exposure of Aβ42 is reduced, resulting in a weakened binding ability of antibodies. Solanezumab, which only binds soluble monomers, recognizes residues 13 to 24 of Aβ42 [26]. Crenezumab recognizes residues 13 to 24 of Aβ42 and has a high affinity for the oligomeric form [26,27,28]. Because of the distinct abilities of these mAbs, corresponding scFvs, scFv-C, scFv-S, and scFv-12B4 were designed and constructed. Their thermal stabilities and binding abilities to Aβ42 were characterized.

The results obtained using Tycho detect the thermal denaturation of proteins based on fluorescence changes during the heating process (from 35 °C to 95 °C) [29]. According to the instructions, during detection, the ratio of 330nm/350nm measures the spectral displacement of Trp residues’ fluorescence emission profile. In the folded state, Trp is usually buried in the hydrophobic nucleus of the protein and exposed to the surface during unfolding. This phenomenon leads to changes in emission intensity and the emission peak wavelength of sample fluorescence, which appears as an inflection point in the unfolded profile and is termed inflection temperature (Ti) [30,31]. The Ti (°C) was 75.3, 62.5, 65.5, and 66.2 for BSA, scFv-C, scFv-S, and scFv-12B4, respectively. These results confirmed that scFvs were thermally stable, and, thus, scFvs could remain in a relatively stable conformation.

Kd represents the affinity of a compound to its target, and the smaller the value, the stronger the affinity [19]. The results from the ELISA showed that scFvs had a high affinity to Aβ42. Ultsch et al. used surface plasmon resonance to detect the affinity of Crenezumab for Aβ42 monomers. The full-length IgG4 exhibited a Kd range of 3.0–5.0 nM for Aβ42 monomers [11]. The results of this study indicated that scFv-C had a better binding ability than scFv-12B4 or scFv-S. ScFv-C also showed a more vital binding ability to Aβ42 monomer than Crenezumab did.

To our knowledge, Crenezumab remains the only antibody that targets the mid-region of Aβ42 peptide and binds to multiple aggregated forms with dissociating effects [16]. The binding ability of scFv-C is the strongest among the scFvs; furthermore, scFv-C disaggregates aggregated Aβ42. Therefore, scFv-C was chosen for further molecular simulations to gain insight into this capability.

Molecular dynamics simulations of scFv-C-Aβ_5_ were performed to obtain more information than the static X-ray crystal structure [15]. The RMSD of backbone atoms was calculated to evaluate the simulation’s convergence to equilibrium and structural stability [32]. The more significant the RMSD is, the greater the deviation of the target molecule from the reference molecule. The RMSD values of the Ala21 to Gly29 region of mono1, mono3, and mono4 of Aβ_5_ were significantly increased by antibody binding, indicating that scFv-C may have the potential to disrupt the structure of Aβ_5_. In addition, it can be observed in Figure 4 that after incubation for 23 h, the fluorescence intensity of the Aβ fiber mixture treated with scFv-C decreases to 39%, which can also imply the deaggregation effect of scFv-C on the formed Aβ42 fiber.

We measured the secondary structure distribution in the scFv-C-Aβ_5_ and Aβ_5_ systems. In the system of scFv-C-Aβ_5_, the Ala21 to Gly29 region of the Aβ_5_ adopts diverse structures, including turn structure, bend structure, 3-helical structure, and random coil. Since helical structures are highly distributed only in soluble Aβ species, this may explain the ability of scFv-C to recognize soluble Aβ42 monomers.

Aβ42 is one of the essential substances that cause AD. ScFvs have a high affinity for Aβ42, and the Kd value of scFv-C is about 2.0 × 10^−9^ M for Aβ42. In addition, scFv-C conformation has specific thermal stability and maintains good stability and activity in the range of human body temperature. A molecular simulation experiment was performed to explore the binding ability of scFv-C and Aβ42 pentamer from multiple perspectives. ScFv-C can bind to monomers, oligomers, and fibrous Aβ42. After binding, there is a tendency for it to depolymerize aggregated Aβ42. In addition, ScFv-C forms hydrogen bonds and salt bonds to prevent Aβ42 from further aggregation. This further provides insight into its potential role in AD. In future experiments, scFv-C might be employed to treat AD model mice of different ages and explore the effects of scFv-C on the nervous system.

## 4. Materials and Methods

### 4.1. Materials

Aβ42 was synthesized by GL Biochem (Shanghai, China). Recombinant plasmids were synthesized by GenScript (Nanjing, China). BL21(DE3) competent cells were purchased from TransGen Biotech (Beijing, China). 3,3,5,5′-tetramethylbenzidine (TMB) was purchased from Tiangen Biotech (Beijing, China). Nitrocellulose membrane was purchased from Whatman (Maidstone, UK). Non-pre-stained Protein Marker was purchased from Thermo Fisher (Rockford, USA). Horseradish peroxidase-labeled (HRP-labeled) goat anti-human IgG (H + L) was purchased from Sino Biological (Beijing, Chian). Anti-His-tag monoclonal antibody was purchased from Invitrogen (Carlsbad, CA, USA). Protein Marker was purchased from Bio-Rad (CA, USA). Mouse monoclonal antibody, β-Amyloid (B-4), recognizing Aβ42 was purchased from Santa Cruz Biotechnology, INC.

### 4.2. Methods

#### 4.2.1. Construction of scFv Plasmids (pscFvs)

Amino acid sequences of scFvs were searched from the Research Collaboratory of the Structural Bioinformatics Protein Data Bank (RCSB PDB). The sequences of scFvs are shown in Table S1. Crenezumab (PDB code 5VZY), Solanezumab (PDB code 4XXD), and 12B4 (PDB code 3IFP) could target the residues from 13 to 24, from 13 to 28, and from 3 to 7 of Aβ42, respectively. The light-chain variable region (VL) and heavy-chain variable region (VH) are connected by a linker, (-Gly-Gly-Gly-Gly-Ser-)_3_. The restriction sites of Xho I and Nco I were constructed on the pET28a vector. The recombinant plasmids were optimized and synthesized by Shanghai Kingsley Company.

#### 4.2.2. Prokaryotic Expression of scFvs

The recombinant pscFvs were transformed into BL21(DE3) competent cells, and a single colony was selected and inoculated overnight in 3 mL of Luria-Bertani (LB) medium containing kanamycin (30 μg/mL) at 37 °C with constant agitation (220 rpm). The next day, these growths were transferred into 30 mL of LB medium and grown to an optical density (OD) of 0.6–0.8 at 600 nm. Isopropyl-β-d-thiogalactopyranoside (IPTG) was added to a final concentration of 1 mM. The samples were harvested after induction culture at 37 °C for 4 h. At the same time, bacteria without induction were broken by ultrasound and centrifuged. The supernatant and precipitate were collected and identified using sodium dodecyl sulfate–polyacrylamide gel electrophoresis (SDS-PAGE).

#### 4.2.3. Purification of Recombinant scFvs

The supernatant was discarded by centrifugation to collect the precipitate, resuspended in 20 mL of 8 M urea (0.05 M Tris, 0.15 M NaCl, pH 8.0, and 8 M urea). After ultrasonic crushing, inclusion bodies were collected by centrifugation at 10,000 r/min at 4 °C for 20 min, and the supernatant was discarded. The inclusion bodies were dissolved in 8 M urea. After centrifugation at 10,000 rpm and 4 °C for 20 min, the supernatant was filtered using a 0.45 μm filter and purified using an affinity His-Trap™ column (GE Healthcare, Piscataway, NJ, USA). The proteins were eluted using imidazole, dialyzed, and concentrated. Finally, the purified proteins were obtained and stored at −20 °C.

#### 4.2.4. Characterization of scFvs Based on Western Blotting

ScFv purity was assessed using SDS-PAGE. The samples were boiled in a SDS sample-loading buffer containing dithiothreitol. The ScFvs were electrophoresed on a 12% SDS-PAGE gel, followed by blotting, and then analysis was performed using Western blotting with an anti-His-tag monoclonal antibody (Invitrogen, Carlsbad, CA, USA). Western blotting was visualized using a ECL Plus substrate (Tanon Science and Technology, Shanghai, China).

#### 4.2.5. Characterization of scFvs’ Binding Activities Based on Enzyme-Linked Immunosorbent Assay (ELISA)

ScFvs were diluted from 4 μM to 1.28 nM in a 1:5 dilution ratio. The ELISA was performed in 96-well plates coated with scFvs (100 μL/well) at 4 °C overnight. Each well was added with Aβ42 to 3 μM. After incubating at 37 °C for 1 h, the plates were washed three times with PBST. β-Amyloid (B-4) (1:2000) was added to the wells for 2 h at 37 °C. After washing with PBST, HRP-labeled goat anti-human IgG (H+L) (1:4000) was added into each well. After incubating at 37 °C for 1 h, 3,3′,5,5′-tetramethylbenzidine solution (Tiangen Biotech, Beijing, China) was added to develop color, and color development was stopped using 2 M H_2_SO_4_. The absorbance at 450 nm was measured using an iMarK™ Microplate Reader (Bio-TEK, Winooski, VT, USA).

#### 4.2.6. Characterization of scFvs’ Thermal Stability

The thermal stability of scFv-C, scFv-S, and scFv-12B4 was detected using Tycho NT.6 (NanoTemper Technologies, Munich, Germany). Bovine serum albumin (BSA) was used as a control.

#### 4.2.7. Promoting Effect on Depolymerization of Aggregated Aβ42

Aggregated Aβ42 was prepared as we previously described [33]. A total of 3 μL of aggregated Aβ42 (1 mg/mL in 10 mM NaOH) and 1.6 μL of Thioflavin-T (THT) solution (5 mM) was mixed in opaque 96-well plates overnight, and aggregated Aβ42 was allowed to form. The ScFvs were mixed in, and the plates were filled to 150 μL with a buffer (pH 7.0) containing 10 mM phosphate buffer (PB) and 500 mM NaCl. Fluorescence was detected every hour using a fluorescence microplate reader (Thermo Fisher Scientific, Waltham, MA, USA) at an excitation/emission wavelength of 485 nm/535 nm, respectively [33].

#### 4.2.8. Three-Dimensional Structure Construction of scFv-C and Aβ42 Pentamer (Aβ_5_) Complex System

In order to predict the potential mechanism of binding between scFv-C and Aβ_5_, the 3D structure of scFv-C was constructed using the Swiss Model web platform [32,34,35,36,37]. The template for homology modeling was selected from the RCSB PDB with the ID of 3AUV [38]. The complex containing scFv-C and Aβ_5_ was obtained using the HADDOCK docking program [39]. According to the position of the complementarity-determining regions (CDR) of the antibody in the template structure, the docking binding-site residues were determined as CDR-L1 (ssqslvysn), CDR-L2 (qlliyvs), CDR-L3 (thvp), CDR-H1 (gftfssygmswv), CDR-H2 (ggst), and CDR-H3 (ycasgdyw). Aβ_5_ was selected to participate in docking with scFv-C. The structure of the scFv-C and its comparison with the template is shown in Figure S1.

#### 4.2.9. Molecular Dynamics Simulation

Molecular simulation sampling was carried out for the scFv-C-Aβ_5_ complex systems and individual Aβ_5_ systems, respectively. The complex systems were subjected to molecular dynamics simulation with periodic boundary conditions using the Gromacs 5.1.5 software package with a simple point-charge water model [40,41,42]. The Gromos 54 A7 force field was applied to describe scFv-C and Aβ_5_ [43].

First, the energies of the complex systems were relaxed with steepest-descent energy minimization to eliminate steric clashes or incorrect geometry. After that, 100 ps NVT (constant Number of particles, Volume, and Temperature) and NPT (constant Number of particles, Pressure, and Temperature) were alternately operated with position restraints on scFv-C and Aβ_5_ to relax the solvent molecules in two phases [44,45]. The solvent molecules were equilibrated with a fixed protein at 310 K, and the initial velocities were chosen from a Maxwellian distribution. Subsequently, scFv-C and Aβ_5_ were relaxed stepwise and heated to 310 K. The long-range electrostatic interactions were described using the particle mesh Ewald algorithm, with an interpolation order of 4, a grid spacing of 0.16 nm, and a Coulomb cutoff distance of 1.0 nm [46]. Temperature-and-pressure coupling types were set using V-rescale and the Parrinello–Rahman method, respectively [47]. In the NVT ensemble, the temperature of the systems reached a plateau at the desired value (reference temperature = 310 K; time constant = 0.1 ps). In addition, the equilibration of pressure (reference pressure = 1.0 bar; time constant = 2.0 ps) was performed under the NPT ensemble. The equilibrated ensembles were subjected to molecular dynamics simulations conducted for 200 ns employing the LINear Constraint Solver (LINCS) and SETTLE algorithm for bond constraints and geometry of water molecules. The 200 ns molecular dynamics simulations were initiated for collecting data with a time step of 2 fs, and coordinates were saved every 2 ps [48,49].

#### 4.2.10. Binding Energy Calculation

The molecular mechanics Poisson–Boltzmann surface area method (MM/PBSA) is applied as a scoring function in computational drug design to estimate free energies in biomolecular interactions [50,51,52]. Using the command “gmx_mmpbsa”, the binding free energy of a complex was calculated from 200 snapshots extracted from the 200 ns MD trajectory. Furthermore, the binding energy was decomposed on a per-residue basis to analyze the individual energy contributions of each residue to the scFv-C-Aβ_5_ interaction.

## Figures and Tables

**Table 1:** The hydrogen bonding probability between scFvs and Aβ_5_.

Donor	Acceptor	Probability
ASN58^scFv-C^-Sidechain	GLU22^Aβ5-mono2^-Sidechain	12.87%
SER72^scFv-C^-Sidechain	GLU22^Aβ5-mono4^-Sidechain	7.92%
THR36^scFv-C^-Sidechain	GLU22^Aβ5-mono4^-Sidechain	7.92%
ASN178^scFv-C^-Sidechain	GLN15^Aβ5-mono5^-Sidechain	22.77%
SER179^scFv-C^-Sidechain	GLN15^Aβ5-mono4^-Sidechain	7.43%
ASN180^scFv-C^-Sidechain	HIS13^Aβ5-mono4^-Sidechain	13.86%
GLY73^scFv-C^-Mainchain	GLU22^Aβ5-mono4^-Sidechain	5.45%
TYR185^scFv-C^-Sidechain	GLN15^Aβ5-mono5^-Sidechain	9.41%
HIS13^Aβ5-mono5^-Sidechain	SER179^scFv-C^-Sidechain	9.90%
TYR37^scFv-C^-Sidechain	VAL18^Aβ5-mono3^-Mainchain	5.45%
SER57^scFv-C^-Sidechain	PHE20^Aβ5-mono2^-Mainchain	11.88%
GLY71^scFv-C^-Mainchain	GLU22^Aβ5-mono3^-Sidechain	9.41%
ASN33^scFv-C^-Sidechain	GLU22^Aβ5-mono4^-Sidechain	9.41%

## Data Availability

The authors confirm that the data supporting the findings of this study are available within the article and its Supplementary Materials.

## Supplementary Materials

The supporting information can be downloaded at https://www.mdpi.com/article/10.3390/ijms24098371/s1.

## Abbreviation

amyloid beta protein (Aβ); amino acid 1–42 of amyloid-β-42 (Aβ42); Aβ42 oligomer (AβO); Aβ42 pentamer (Aβ_5_); Alzheimer’s disease (AD); thermophilic acylpeptide hydrolase (APH); amyloid-related imaging abnormalities (ARIA); bovine serum albumin (BSA); complementarity-determining regions (CDR); crystallizable fraction (Fc); horseradish peroxidase-labeled (HRP-labeled); isopropyl-β-d-thiogalactopyranoside (IPTG); equilibrium dissociation constant (Kd); Luria-Bertani (LB); LINear Constraint Solver (LINCS); monoclonal antibody (mAb); molecular dynamics (MD); molecular mechanics Poisson–Boltzmann surface area method (MM/PBSA); constant Number of particles, Pressure, and Temperature (NPT); constant Number of particles, Volume, and Temperature (NVT); presenilin 1 (PSEN1); phosphate-buffered saline (PBS); PBS containing 0.5 percent Tween 20 (PBST); Research Collaboratory for Structural Bioinformatics (RCSB); single-chain variable fragment (scFv); Crenezumab-like scFv (scFv-C); Solanezumab-like scFv (scFv-S); 12B4-like scFv (scFv-12B4); sodium dodecyl sulfate–polyacrylamide gel electrophoresis (SDS-PAGE); Thioflavin-T (THT); inflection temperature (Ti); 3,3,5,5′-tetramethylbenzidine (TMB); tryptophan (Trp).
