# Supplementary material for: Mechanism Exploration of Amyloid-β-42 Disaggregation by Single-Chain Variable Fragments of Alzheimer’s Disease Therapeutic Antibodies
Source: Int J Mol Sci. 2023 May 6;24(9):8371. doi: 10.3390/ijms24098371 (PMC10179127; doi:10.3390/ijms24098371)
Supplement: Supplementary file 1 [file ijms-24-08371-s001.zip › ijms-2336116-supplementary.pdf]

Table S1 The sequences of scFvs with light and heavy chains connected by (-Gly-Gly-Gly-Gly-Ser-)$_3$ in the variable fragment regions of antibodies

scFvs	Sequences
scFv-C	divmtqsplslpvtpgepasiscrssqslvysngdtylhwylqkpgqspqlliykvsnrfsgvpd<br>rfsgsgsgtdftlkisrveaedvgvyycsqsthvpwtfgqgtkveiGGGGSGGGGSGGG<br>GSevqlvesggglvqpggslrlscaasgftfssygmswvrqapgkglelvasinsnggstyypd<br>svkgrftisrdnaknslylqmnslraedtavyycasgdywgqgttvtvsGGGGSGGGGS<br>GGGGSHHHHHH
scFv-S	dvvmtqsplslpvtlgqpasiscrssqsliysdgnaylhwflqkpgqsprlliykvsnrfsgvpdr<br>fsgsgsgtdftlkisrveaedvgvyycsqsthvpwtfgqgtkveikrtGGGGSGGGGSGG<br>GGSevqlvesggglvqpggslrlscaasgftfsrysmswvrqapgkglelvaqinsvgsstyyp<br>dtvkgrftisrdnakntlylqmnslraedtavyycasgdywgqgtlvtvssasGGGGSGGG<br>GSGGGGSHHHHHH
scFv-12B4	dvlmtqtplslpvslgdqasiscrssqnivhsngntylewylqkpgqspklliykvsnrfsgvpd<br>rfsgsgsgtdftlkisrveaedlgvyycfqgshvpltfgagtklelkGGGGSGGGGSGGGG<br>Sqvtlkesgpgilqpsqtlsltcsfsgfslstngmgvswirqpsgkglewlahiywdedkrynps<br>lksrltiskdtsnnqvflkitnvdtadtatyycarrriiydvedyfdywgqgttltvssGGGGSG<br>GGGSGGGGSHHHHHH

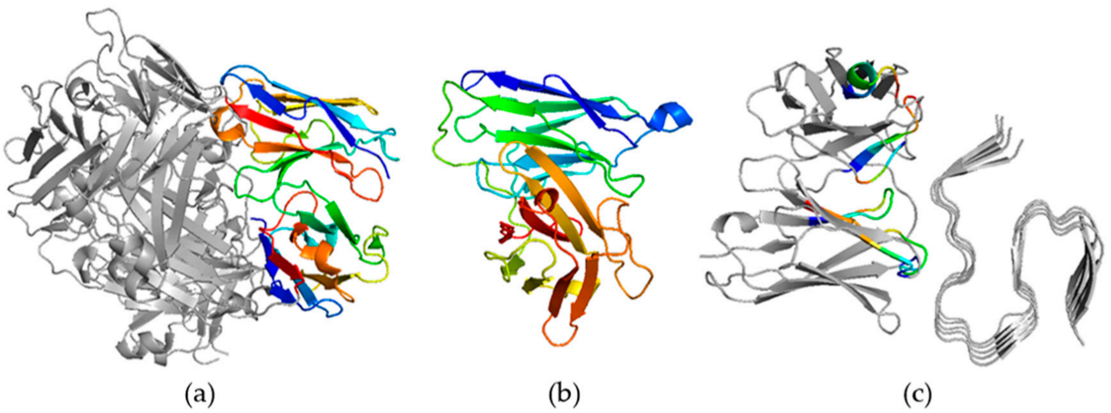

Figure S1 Structural comparison of scFv-C and Crenezumab. (a) The structure of Crenezumab. (b) The structure of scFv-C. (c) scFv-C and Aβ_5 composite structure.

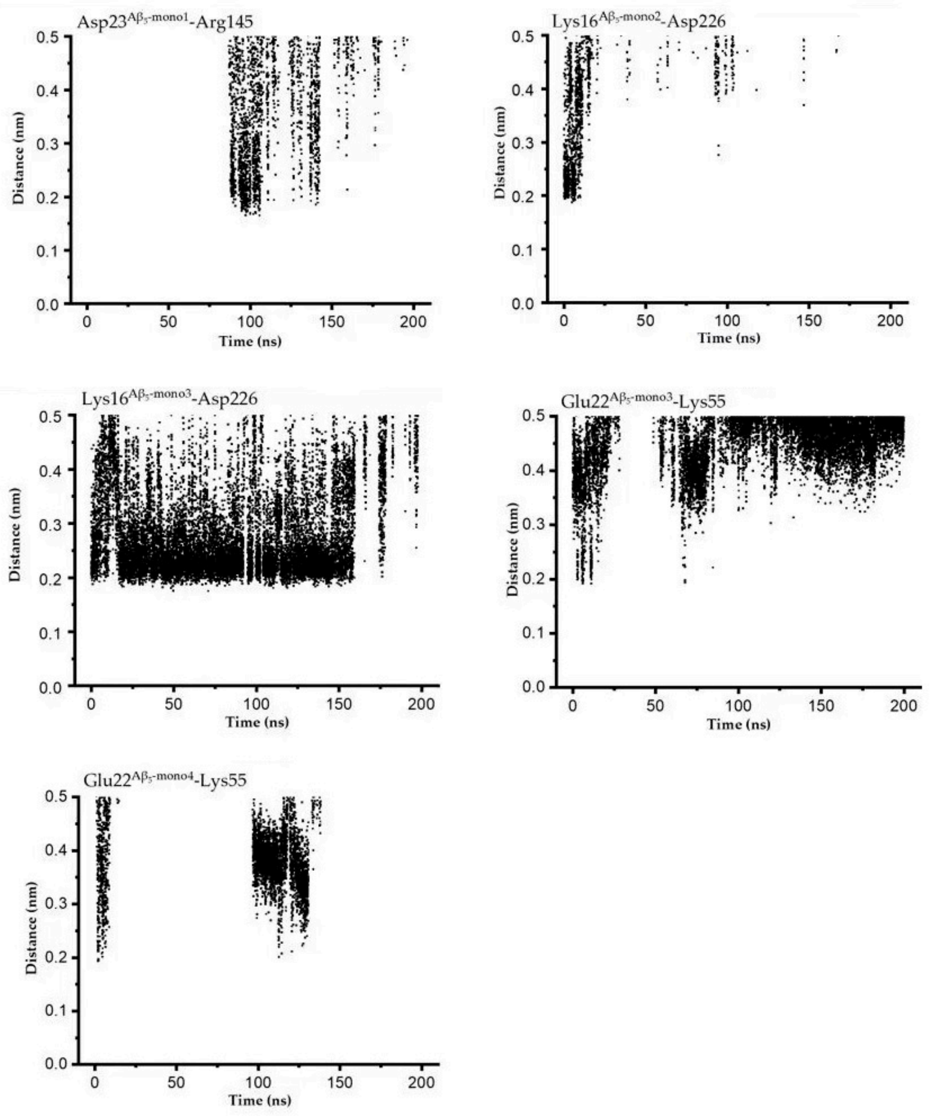

Figure S2 The distances of charged residues at the binding interface of scFv-C with Aβ_5 complex.
